# Unconscious processing of facial attractiveness: invisible attractive faces orient visual attention

**DOI:** 10.1038/srep37117

**Published:** 2016-11-16

**Authors:** Shao-Min Hung, Chih-Hsuan Nieh, Po-Jang Hsieh

**Affiliations:** 1 Neuroscience and Behavioral Disorders Program, Duke-NUS Medical School, Singapore; 2 Department of Psychology, National University of Singapore, Singapore.

## Abstract

Past research has proven human’s extraordinary ability to extract information from a face in the blink of an eye, including its emotion, gaze direction, and attractiveness. However, it remains elusive whether facial attractiveness can be processed and influences our behaviors in the complete absence of conscious awareness. Here we demonstrate unconscious processing of facial attractiveness with three distinct approaches. In Experiment 1, the time taken for faces to break interocular suppression was measured. The results showed that attractive faces enjoyed the privilege of breaking suppression and reaching consciousness earlier. In Experiment 2, we further showed that attractive faces had lower visibility thresholds, again suggesting that facial attractiveness could be processed more easily to reach consciousness. Crucially, in Experiment 3, a significant decrease of accuracy on an orientation discrimination task subsequent to an invisible attractive face showed that attractive faces, albeit suppressed and invisible, still exerted an effect by orienting attention. Taken together, for the first time, we show that facial attractiveness can be processed in the complete absence of consciousness, and an unconscious attractive face is still capable of directing our attention.

Nearly 100 years ago, Thorndike^1^ has pointed out that the ratings of separate personal traits were influenced by the general impression of the person, namely the “Halo effect”. The Halo effect has paved the path to explain the potency of attractiveness: The more attractive one is conceived, the more positive other personal traits are judged. In fact, being appraised as an attractive person or not plays an important role in one’s life, influencing how one is judged^2^,^3^,^4^, how likely one can be hired after an interview^5^, and even how easily an infant catches attention from his/her mother^6^.

It is a common belief that our conception of beauty results from a mixture of vivid sensation (what we perceive consciously) and close inspection (what we scrutinize): attractiveness does not simply impinge on our retina and reflect itself, it needs to be *seen* and *appreciated*. Thus, to judge the attractiveness of a piece of artwork, a scene, and even a face seems to require conscious deliberation. However, previous literature has shown human’s extraordinary ability to extract facial attractiveness in extreme cases, making little room for deliberation. Firstly, trait judgments on faces including attractiveness without time constraints correlated significantly with those made after only a 100-ms exposure, showing that first impressions could be formed in the blink of an eye^7^. Secondly, infants that were too little to be confined to our social norm (i.e. less than 3 month olds) seem to have a similar standard of attractiveness^8^, suggesting an attractiveness detector is well functioning early in development. Furthermore, it has been shown that even when faces were presented for an extremely brief of time (i.e. 13 ms) so that their identity can not be consciously perceived, participants could still distinguish their attractiveness^9^. This line of work has demonstrated that facial attractiveness can be extracted with minimal conscious endeavour, directing us to a counterintuitive hypothesis: attractiveness judgment can be made automatically and unconsciously. Nevertheless, it has never been examined if one could process facial attractiveness in the complete absence of consciousness.

Here we directly tested this conundrum in a series of experiments with different approaches. We hypothesized that facial attractiveness could be extracted and even direct attention without one having visual awareness of a face. Continuous flash suppression^10^ was utilized to ensure longer suppression time and stronger suppressive power to abolish not just identification but also any visibility of the face, which is the major difference between this interocular suppression and other masking techniques where participants might still be conscious of parts of the stimulus (e.g. crowding^11^; masking^12^). Therefore, being unconscious of a face in all our experiments denotes not seeing any part of the face but not just “failure to identify” the faces. In Experiment 1, the faces were suppressed and the time taken to perceive the faces were measured (*suppression time*). Importantly, the attractiveness of the faces was rated by each individual participant in a surprise task after the main experiment, so that attractive and unattractive faces can be sorted and analyzed according to each individual’s own evaluation. In Experiment 2, we turned to measure the visibility threshold of the faces under suppression rather than the time taken to break suppression. In Experiment 3, to further make the claim that facial attractiveness can be processed unconsciously and an unconscious attractive face still exerts the benefit of orienting one’s attention, we examined only the trials where faces were reported invisible. Participants judged the orientation of a tilted Gabor patch presented subsequent to a suppressed/invisible attractive or unattractive face. If facial attractiveness can be extracted even from an unconscious face, we expect to see that the suppression time, visibility threshold, and post-face performance on the Gabor orientation discrimination differ between attractive and unattractive unconscious face conditions.

## Methods

### Experiment 1

#### Participants

Twenty-nine participants (age range: 18–35) were recruited for the experiment. In this and all the subsequent experiments, all participants reported normal or corrected-to-normal vision. All participants gave informed consent prior to the experiment and were reimbursed $10 for a session lasting 60 minutes. All experiments in this study were approved by the NUS Institutional Review Board, and the methods were performed in accordance with relevant guidelines and regulations. One participant was excluded due to low performance accuracy on the location task (see below). One participant reported feeling strong emotions from the face stimuli was also excluded. Seven participants were excluded due to neutral ratings (see rating). Twenty participants (10 males) were included in the final analysis, this criterion was set according to previous studies on unconscious face processing^13^,^14^. We recruited equal number of male and female participants and set out to examine whether the gender of the participants and the face stimuli interact.

#### Stimuli and procedures

**Figure 1:**
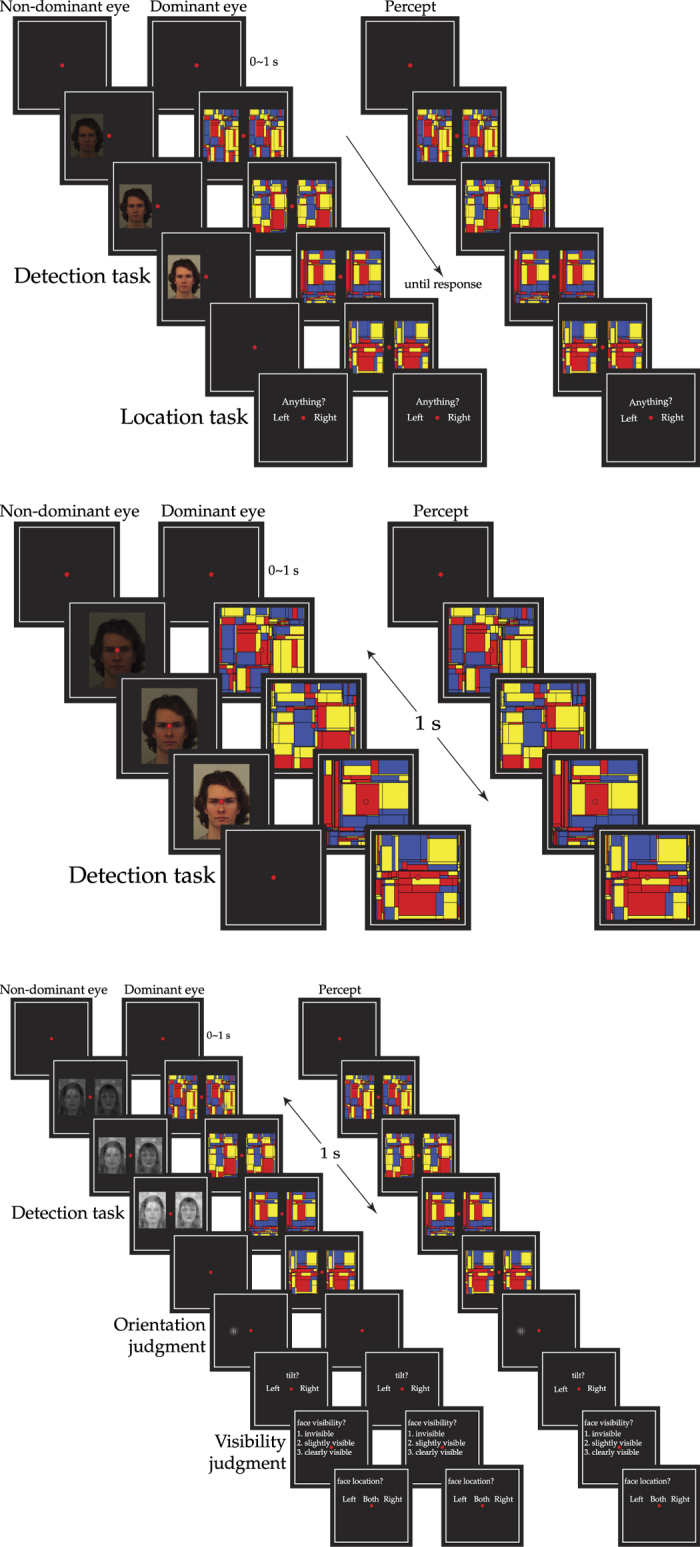
Procedure and stimuli in Experiments 1 (top), 2 (middle), & 3 (bottom). Colorful Mondrians were always presented on the dominant eye to ensure strong suppressive strength in all experiments. In Experiment 1, alpha of target increased from 0–75% over 10 seconds, either on the left or right of the fixation point. Participants were required to press a button when targets became visible (Detection task), and then answered the validity question (Location task). Time-to-detection (i.e. *suppression time*) was the dependent variable. In Experiment 2, alpha of target ramped up to designate value in one second. Participants were required to press a button when targets became visible (Detection task). Target face contrast was changed adaptively according to the response (detected/non-detected) throughout the experiment with a 1-up-1-down procedure. Contrast-to-detection (i.e. *visibility threshold*) was the dependent variable. In Experiment 3, an attractive face and an unattractive face were presented simultaneously on the left and right, followed by a brief flash (i.e. 100 ms) of a Gabor patch on the left or right. Participants were instructed to report the tilt of the Gabor patch and the visibility of the faces. Gabor orientation discrimination accuracy was compared subsequent to invisible attractive and unattractive faces.

58 faces (29 males) were selected from the Karolinska Directed Emotional Faces (KDEF) database^15^ under the emotionally neutral category. In Experiment 1, each face was presented 4 times, resulting in 232 trials. The experimental procedure is shown in Fig. 1. Each trial began with a fixation period ranging from 0 to 1 second. After that, the dominant eye received two full-contrast dynamic pattern suppressors (a series of colorful Mondrians) flashing at 10 Hz, presented simultaneously in two locations – to the left and right of the fixation point. Meanwhile, the non-dominant eye received a target face, either to the left or right side of the fixation point, with the contrast (alpha value) of the target face ramping up gradually with the speed of 7.5% per second. The target face was invisible at the beginning of the presentation (0% alpha) while the percept was suppressed by the flashing Mondrians presented to the dominance eye until visibility was indicated.

Participants were required to press the space bar when any part of the target face became visible (Detection task), followed by indicating whether it appeared at the left or the right side of the fixation point with left or right arrow key (Location task) (Fig. 1, top figure). The aim of the location task was to catch trials in which participants responded prematurely/incorrectly before seeing the target stimulus. The Mondrians stayed on the screen for 500 ms after visibility was indicated to prevent the participants from seeing an afterimage of the target stimuli or up to ten seconds. In non-broken trials, the target face stayed at the final contrast (i.e. 75%) for 500 ms after the Mondrians were turned off, which ensured the visibility of the target stimulus. Note that in all experiments, participants were not asked to report the identity of the face.

Visual stimuli were generated with MATLAB PsychToolbox. Participants viewed a pair of dichoptic images through a mirror stereoscope mounted on a chin rest, from a distance of 57 cm. Each face subtended 2.05 × 2.80 visual degrees. Stimuli were presented against a black background on a 22” Samsung 2233RZ LCD monitor with a resolution of 1680 × 1050 pixels and a refresh rate of 120 Hz. Throughout the experiments, a white frame (subtending 4.3 × 4.3 visual degrees) remained on screen to facilitate proper fusion. Participants were asked to maintain fixation on a red central fixation point.

After the main continuous flash suppression experiment, participants were told to participate in one extra surprise rating experiment in which all 58 faces used in the main experiment were rated according to their attractiveness on a 8-point scale (from 1 (not attractive at all) to 8 (very attractive)). Participants were told to utilize the full scale. Participants viewed the visual presentation while resting on a chin rest with a distance of 44 cm (no dichoptic presentation used). In each trial, the face was presented for 3 seconds followed by the rating. Face stimuli subtended 16 × 12 visual degrees and were presented against a black background on a 21.5” iMAC LCD monitor with a resolution of 1920 × 1080 pixels and a refresh rate of 60 Hz.

#### Results and Discussion

In the main experiment, participants with accuracy three standard deviations below the group mean for the location task were removed prior to analysis. Also, trials where the location task was answered incorrectly or with suppression time more than three standard deviations above or below the mean of the correspondent condition in each participant were excluded.

We later categorized each face as attractive or unattractive by the individual ratings from each participant. Faces with ratings above 5 were deemed as attractive, whereas faces with ratings below 4 were deemed as unattractive. The mean ratings for attractive and unattractive faces were 5.7 and 2.9 respectively (*t*_paired_(9) = 19.33, *p* < 0.00001). Note that every participant had a different set of attractive and unattractive faces based on his/her subjective judgment.

**Figure 2:**
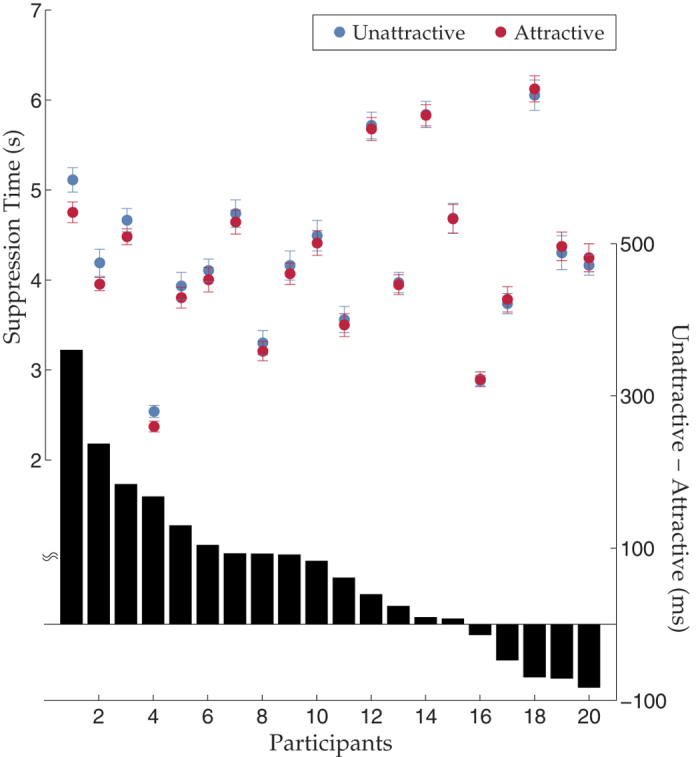
Individual suppression time in Experiment 1. Each column denotes one participant, ranked by strength of the effect. Dots denote suppression time of the attractive (red) and unattractive (blue) conditions (*left y-axis*). Error bars are *SEM.* Bars in the lower part of the figure show suppression time difference (*right y-axis*).

Experiment 1 revealed that attractive faces broke through suppression faster and thus reached consciousness earlier. We performed a 3-way repeated measures ANOVA with gender of the participants as the between-subject factor, and gender and attractiveness of the target faces as the two within-subject factors. The results showed main effects on both the attractiveness (F(1,19) = 17.9, p < 0.001) and gender of the faces (F(1,19) = 5.7, p < 0.05) with no interaction between the two (F(1,19) = 2.3, p > 0.05). Overall, attractive faces broke through CFS significantly faster (Mean suppression time (mean ± standard error of the mean): attractive: 4.24 ± 0.12 s; unattractive: 4.31 ± 0.14 s) (Fig. 2). The face attractiveness effect could not be explained by the face gender effect because the two effects had opposite directionality. That is, overall male faces had lower attractive ratings (male vs. female: 3.5 vs. 4.0, *t*_paired_(19) = −3.21, *p* < 0.05) but shorter suppression time (male vs. female: 4.20 s vs. 4.32 s, *t*_paired_(19) = 2.39, *p* < 0.05). We did not find any significant effect from the gender of the participants (F(1,19) = 0, p > 0.05). Furthermore, we calculated the mean attractiveness rating of each face from the 20 participants and correlated the ratings with suppression time. If across participants there was enough overlap as to which face is attractive/unattractive, then we expected to see a negative correlation between the attractive ratings and suppression time. Indeed, we observed a significant negative correlation between the two, showing that even at the group level, the higher the mean attractiveness rating was given, the faster a face broke through CFS (r = −0.24, *p* < 0.05, Figure S1).

#### Experiment 2

In Experiment 1 we adopted a breaking CFS paradigm (bCFS) in which the time taken for an image to reach consciousness was measured. However, a recent review has pointed out that bCFS might merely measure the speed of access to consciousness but not unconscious processing per se^16^. In order to replicate what we found in Experiment 1 as well as to ensure it was not just a bCFS-specific effect, we turned to measure the *visibility threshold* of attractive and unattractive faces. Because attractive faces enjoyed the privilege to break through suppression earlier in Experiment 1, we hypothesized that attractive faces would have lower visibility thresholds.

##### Participants

Since we did not observe any significant gender effect from the participants in Experiment 1, we set out to recruit 10 participants regardless their gender. Thirteen participants (age range: 18–35) were recruited for Experiment 2. All participants gave informed consent prior to the experiment and were reimbursed $10 for a session lasting 60 minutes. One participant was excluded due to neutral ratings on the attractiveness judgment task. Two participants were excluded due to performance 3 standard deviations away from the group mean. Ten participants (3 males) were included in the final analysis.

##### Stimuli and procedures

We selected the top four attractive faces and four unattractive faces based on the ratings of the participants in Experiment 1 (Mean_attractive_: 5.48 ± 0.12; Mean_unattractive_: 2.23 ± 0.03, *t*_paired_(9) = 14.91, *p* < 0.00001). Also, post-main-experiment surprise rating session in Experiment 2 confirmed significant differences between the two categories (attractive vs. unattractive faces: 5.3 ± 1.1 vs. 2.1 ± 0.6, *t*_paired_(9) = 10.59, *p* < 0.00001; Rating correlation across all faces between Experiments 1 and 2: r = 0.79, p < 0.00001). The contrast (i.e. alpha value) of each face was changed adaptively throughout the experiment with a 1-up-1-down procedure (i.e. The Bruceton test^17^: the contrast decreased or increased by 4% in the next trial when the shape was detected or undetected respectively. Each face had two staircases: one had a starting contrast at 50% (top) and another one at 10% (bottom). Each staircase had 30 trials, resulting in 480 trials (8 faces × 2 staircases × 30 trials). All the staircases were mixed in the experiment, and each trial was randomly chosen from one of the sixteen possible conditions.

The binocular setup was identical to that in Experiment 1. In Experiment 2 each face subtended approximately 2.68 × 3.63 visual degrees. After a SOA (stimulus onset asynchrony between the blank and the suppressor) ranging from 0 to 1 second, the dominant eye received the suppressor flashing at 10 Hz, while the non-dominant eye received the face stimulus with the contrast ramping up gradually to the target contrast in 1 s. The target contrast was determined by participants’ response in the previous trial of the same staircase (i.e. the 1-up-1-down procedure). After the face disappeared, the suppressor remained for 300 ms to prevent any afterimage. Participants were required the indicate visibility any time during the trial if they have detected any part of the face (Fig. 1, middle figure).

##### Results and Discussion

**Figure 3:**
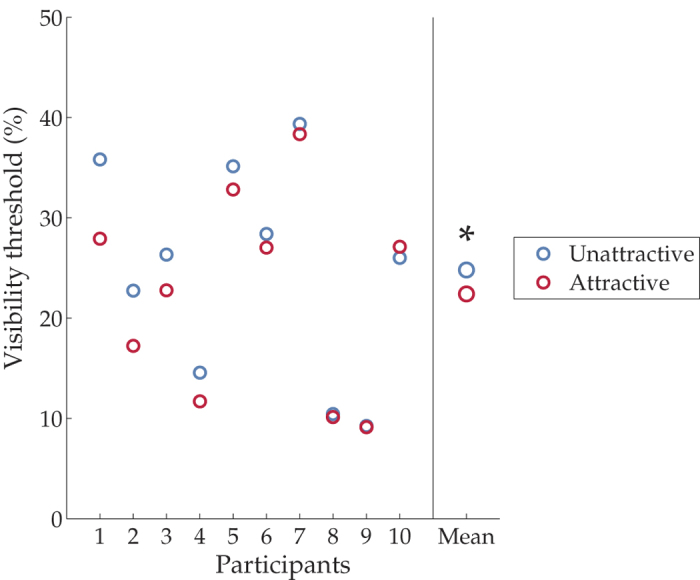
Visibility thresholds (i.e. contrasts) in percentage in Experiment 2. The left panel shows the visibility thresholds in the attractive (red) and unattractive (blue) conditions of individual participants, ranked by strength of the effect. The right panel shows the group mean.

A two-way repeated measures ANOVA was performed with two within-subject factors: attractiveness and gender of the faces. We observed main effects on both factors: attractiveness (F(1,9) = 7.3, *p* < 0.05) (Fig. 3) and gender (F(1,9) = 7.6, *p* < 0.05). Furthermore, there was an interaction between the two (F(1,9) = 6.3, *p* < 0.05). Post-hoc t tests showed that unattractive male faces had significantly higher visibility thresholds (Mean visibility thresholds (mean ± standard error of the mean): 22.0 ± 3.3% v.s. 27.6 ± 4.0%, *t*_paired_(9) = 2.89, *p* = 0.018, significant after Bonferroni correction) (Figure S2). However, male faces were not rated as more unattractive (Mean: female vs. male: 3.73 vs. 3.63. *t*_paired_(9) = 0.29, *p* = 0.78), again suggesting that the gender effect was independent of the attractiveness effect.

#### Experiment 3

In Experiments 1 and 2, we showed that faces evaluated as attractive enjoyed a privilege to reach consciousness easier, reflected by shorter suppression time and lower visibility threshold. However, one question remained unanswered: *Why do unconscious attractive faces enjoy such privilege?* In Experiment 3, we hypothesized that attractive faces, even when suppressed and invisible, are still able to direct our attention. To examine this possibility, we tested how invisible attractive and unattractive faces influence the performance of a subsequent Gabor orientation discrimination task. If invisible faces orient attention differently depending on their level of attractiveness, then subsequent Gabor patch orientation discrimination performance is expected to differ.

##### Participants

Thirteen participants (age range: 18–35) were recruited. All participants gave informed consent prior to the experiment and were reimbursed $10 for a session lasting 60 minutes. One participant was excluded due to neutral ratings on the attractiveness judgment task. Two participants were excluded due to high false alarm rates (see experimental procedure). Ten participants (3 males) were included in the final analysis.

##### Stimuli and procedures

In addition to the attractiveness effect, Experiments 1 and 2 also showed a gender effect in a different direction, suggesting that the gender effect might be a low-level confound (e.g. contrast difference between male and female faces). To control for this potential confounding factor, the color of the face stimuli taken from Experiment 2 were removed and SHINE toolbox^18^ was applied to balance mean luminance and contrast differences. During the experiment, the contrasts of the faces were changed adaptively as we implemented in Experiment 2 except that we turned to a two-up-one-down procedure: the contrast increased in the next trial when invisibility was indicated in the previous two trials consecutively and decreased in the next trial when visibility was indicated in the previous trial. Each face subtended 2.80 × 2.05 visual degrees. The Gabor patch had a spatial frequency of 4 cycles per degree and subtended 1.1 × 1.1 visual degrees.

Prior to each experiment, every participant underwent a calibration session with 120 trials that were identical to the main experiment in which both the orientation of the Gabor and the contrast of the faces were changed adaptively in a staircase procedure. This calibration sought to measure (1) the thresholds of the faces for the two locations (i.e. left and right) and (2) the tilt of the Gabor patch required for each participant that lead to accuracy of orientation discrimination ranging from 70~95%.

Each trial began with a 0 to 1 second fixation period. After which, two series of flashing suppressors were presented to the dominant eye on the left and right side of the fixation point. After 100 ms, on the non-dominant eye, one attractive and one unattractive faces of the same gender were presented for 1 s repetition. The suppressors remained on screen for another 100 ms after the faces disappeared to prevent afterimages. 100 ms after the suppressors disappeared, a Gabor patch was presented on the left or right side (i.e. center of one of the face stimuli) for 100 ms. Participants were instructed to discriminate the orientation of the Gabor patch (left- or right-tilted) immediately, followed by reporting the visibility of the faces. Once face visibility was indicated, participants had to report the location of the visible face(s) (Fig. 1, bottom figure). Note that this procedure was designed to help with threshold adjustment for the locations but not attractive and unattractive faces. Across participants, attractive and unattractive faces were presented at comparable contrasts (Contrast_attractive_ = 34.1%, Contrast_unattractive_ = 34.1%, *t*_paired_(9) = −0.08, *p* > 0.5). The locations of the attractive and unattractive faces and the location and tilt of the Gabor patch were counterbalanced, so that none of the factors were predictive of another factor.

##### Results and Discussion

**Figure 4:**
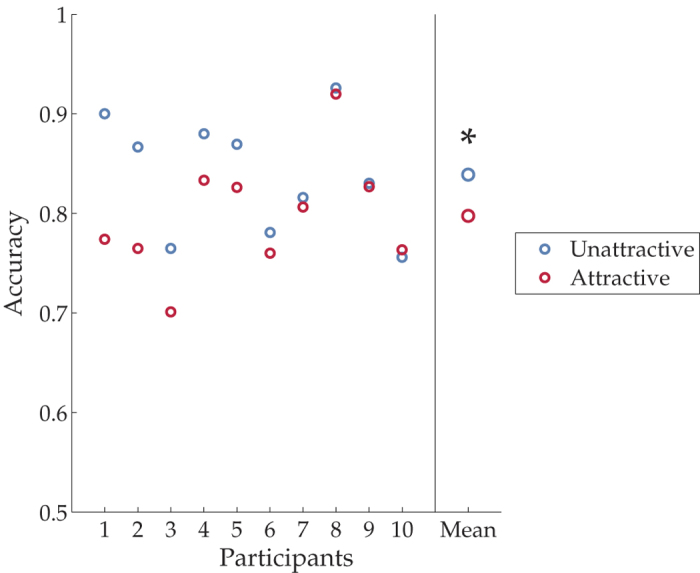
Accuracies on the subsequent orientation discrimination task in Experiment 3. The left panel shows the accuracies subsequent to an attractive (red) or an unattractive (blue) face of individual participants, ranked by strength of the effect. The right panel shows the group mean.

If attractive faces could still attract attention unconsciously, one would expect to see different accuracies of Gabor patch orientation discrimination between the two sides of fixation. Specifically, because the SOA (stimulus onset asynchrony between the faces and a Gabor) between the cue (i.e. invisible faces) and target (i.e. the Gabor) was longer than 300 ms and the cue was not predictive of the target, we expected the effect of inhibition of return^19^, which should lead to a lower accuracy on the “attractive side”. We thus performed a paired t-test to compare the accuracy on the attractive and unattractive sides. Our results showed a clear reduction of accuracy on the attractive side (attractive vs. unattractive (Mean accuracy ± standard error of the mean): 79.76 ± 2.00% vs. 83.89 ± 2.00%, *t*_paired_(9) = −3.53, *p* = 0.007, Fig. 4). We did not observe any face gender effect (t_unpaired_ (14) = 0.78, p > 0.05).

## General Discussion

Our results show that attractiveness can be processed in the complete absence of conscious awareness. This unconscious effect was shown with different measurements in three experiments. Firstly, Experiment 1 showed that the attractiveness was processed prior to consciousness in that attractive faces broke through suppression faster and thus reached consciousness earlier. Similarly, this effect was shown with a thresholding paradigm in Experiment 2: attractive faces exhibited lower visibility thresholds, suggesting that facial attractiveness was processed more easily prior to consciousness, namely in a “pre-conscious” manner. Crucially, Experiment 3 demonstrated that unconscious attractive faces still exerted the effect of orienting attention, reflected by a significant drop of accuracy on the subsequent orientation discrimination task. Taken together, we show that conscious perception is not a necessary condition for facial attractiveness to be processed, and an unconscious attractive face still yielded potent effect by directing our attention.

Our findings suggest that an integration of facial components exists outside of the realm of consciousness. This object-level integration has been suggested when target is blocked from recognition with different masking techniques, such as backward masking^20^ and crowding^21^. Under continuous flash suppression, several recent studies have shown that familiarity and the emotions of the faces can be processed unconsciously. For example, it has been shown that upright faces break through interocular suppression faster than inverted faces, possibly due to familiarity^13^. Along the same line, a recent study has shown an advantage of detecting familiar faces (e.g. friends) under interocular suppression^22^. Also, fearful faces, compared to neutral and happy faces, were shown to gain consciousness earlier during interocular suppression^14^. However, at least one recent study has pointed out the possibility of low-level confound: face emotion effect found under CFS might be due to local face features but not result from high-level integration^23^. In the current study, apart from controlling for low-level features of the faces in Experiment 3 (See Experiment 3, *stimuli and procedure*), we further conducted a control experiment of Experiment 3 with half of the trials containing upright faces and the other half containing inverted faces. In addition to replicating what we found in Experiment 3 with upright faces, the unconscious effect clearly disappeared in the inverted face condition, suggesting that the unconscious attractiveness effect was likely due to integration of face features but not driven by local or lower-level features (see supplementary text and Figure S3).

Our Experiment 3 further pointed out one possible explanation of the “unconscious attractiveness benefit”: *attention*. It has been shown that a simple and salient stimulus could attract one’s attention unconsciously: a feature singleton (i.e. Gabor patch) is able to attract attention when presented alone^24^ or in a set of homogeneous distractors, namely an unconscious pop-out effect^25^,^26^. Our Experiment 3, on the other hand, demonstrated feature-integration-driven attention could come into play even when the stimulus was well suppressed and wiped out from conscious awareness. Undoubtedly, there was an unconscious processing of facial attractiveness in Experiment 3, leading to distinct performance on the orientation discrimination between a Gabor patch temporally and spatially subsequent to an attractive and an unattractive face. But, why did we observe an inhibition of accuracy subsequent to an unconscious attractive face? Our results might exhibit the classical inhibition of return (IOR) of an exogenous cue. IOR was first found by Posner & Cohen^27^ and attributed to disengagement of attention to the cued location after a long delay (stimulus onset asynchrony (SOA) between a cue and a target), allowing further orientation to new locations (for an extensive review on IOR, see ref. 19). This inhibition effect has been shown with conscious faces^28^ and unconscious simple cues^29^. Our results are in line with this account, showing that a prolonged and uninformative cue (i.e. our faces were not predictive of the location of the cue) actually harms the performance on a following task (but see ref. 30). However, we observed a decrease in accuracy but not a typical delayed reaction time to the target, which might be because the two demanding tasks (i.e. face detection and orientation discrimination) were interfering with each other. In other words, it was demanding that participants had to fixate in the center while constantly searching for any broken stimulus during the prolonged presentation of the unconscious faces. Future experiments are needed to show whether delayed reaction time can be observed with reduced task demand.

Overall, our findings support that, similar to other features that an be processed unconsciously^24^,^31^,^32^, face processing is also extremely automatic: facial attractiveness can be extracted and even direct one’s attention when the faces were under strong interocular suppression and thus rendered invisible. Indeed, if conscious awareness is not a prerequisite to extract facial attractiveness, our behavior might be even more susceptible to the attractiveness of a stimulus than we once believed, offering another reason why the conception of beauty has such a profound impact on human society.

## Additional Information

**How to cite this article**: Hung, S.-M. *et al.* Unconscious processing of facial attractiveness: invisible attractive faces orient visual attention. *Sci. Rep.* **6**, 37117; doi: 10.1038/srep37117 (2016).

**Publisher's note**: Springer Nature remains neutral with regard to jurisdictional claims in published maps and institutional affiliations.

## Supplementary Material

Supplementary Information
