# Supplementary material for: Unconscious processing of facial attractiveness: invisible attractive faces orient visual attention
Source: Sci Rep. 2016 Nov 16;6:37117. doi: 10.1038/srep37117 (PMC5111056; doi:10.1038/srep37117)
Supplement: Supplementary Information [file srep37117-s1.pdf]

Unconscious processing of facial attractiveness: invisible attractive faces orient visual attention.

Shao-Min Hung[1], Chih-Hsuan Nieh[2], & Po-Jang Hsieh[1*]

[1]Neuroscience and Behavioral Disorders Program, Duke-NUS Medical School, Singapore

[2]Department of Psychology, National University of Singapore, Singapore

[*]*Corresponding author:* Po-Jang Hsieh (pojang.hsieh@duke-nus.edu.sg), Neuroscience and Behavioral Disorders Program, Duke-NUS Medical School, 8 College Road, 169857 Singapore

Supplementary text

1. **Correlation between attractiveness ratings and suppression in Experiment 1**

2. **Interaction between face gender and attractiveness in Experiment 2**

3. **Control Experiment of Experiment 3**

1. **Negative correlation between attractiveness ratings and suppression time across all participants in Experiment 1**

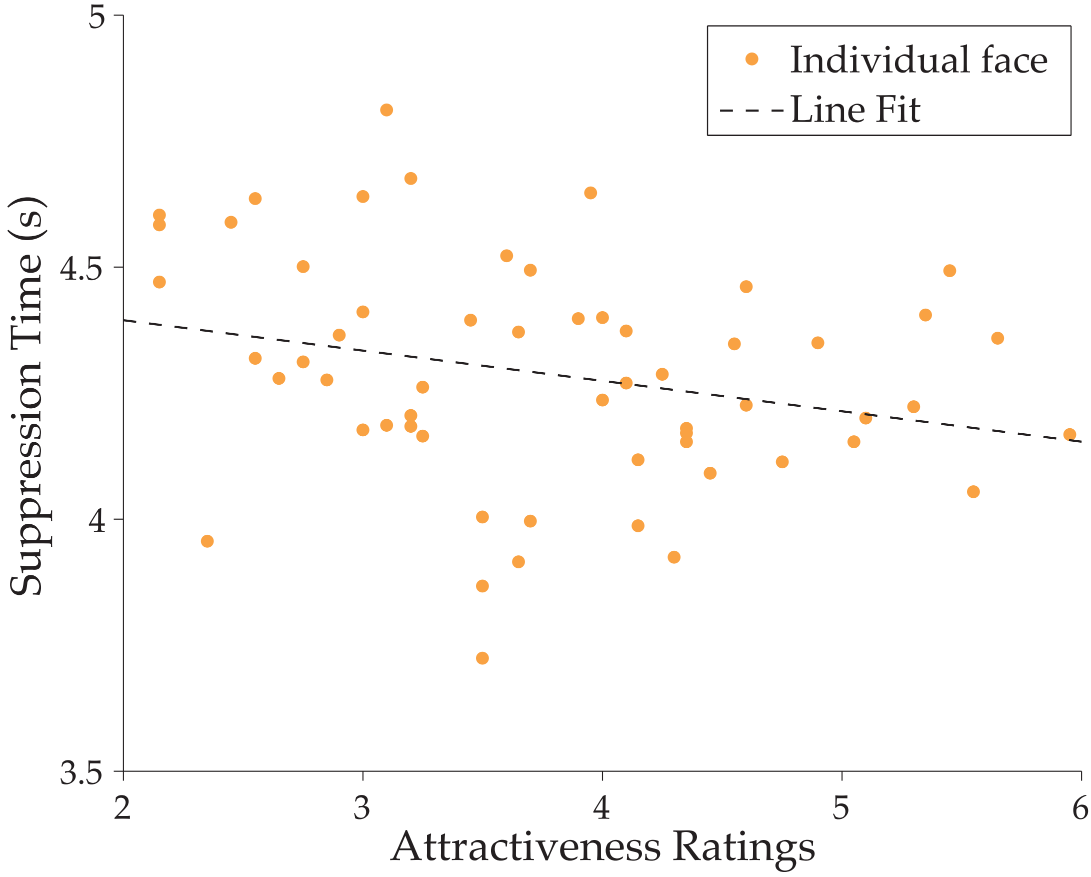

Figure S1. Correlation between attractiveness ratings and suppression time across participants (r = -0.24, $p < .05$). Each dot denotes the mean suppression time and attractiveness ratings of one of the 58 faces across twenty participants.

2. Interaction between face gender and attractiveness in Experiment 2

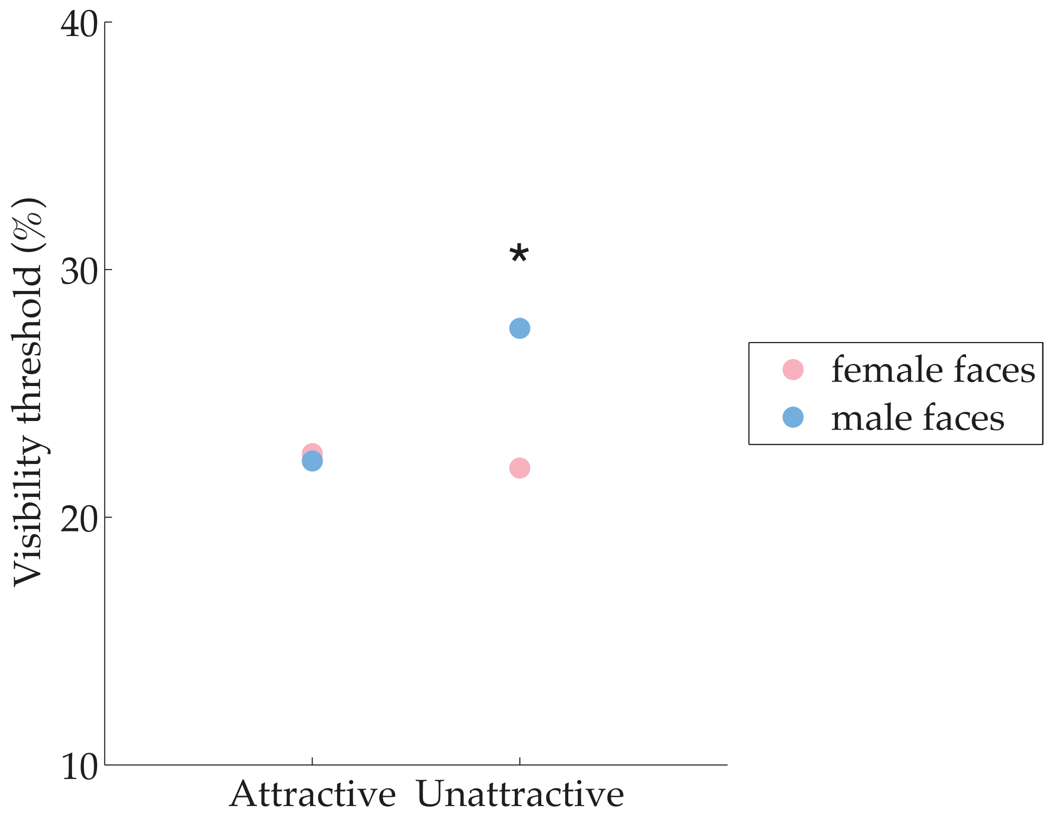

Figure S2. A face attractiveness and gender interaction on visibility threshold in Experiment 2. Post-hoc t tests showed that unattractive male faces had significantly higher visibility thresholds.

3. Control experiment of Experiment 3

Although we have controlled for mean luminance and contrast differences by making the faces black-and-white and applying the SHINE toolbox in Experiment 3, one could still argue that other low-level image features (e.g. hair volume) might still drive the effect and confound our results. We have thus conducted a control experiment with identical setup and procedure as Experiment 3 and recruited another 10 participants (4 males, age range: 18-35, all participants gave informed consent prior to the experiment and were reimbursed $10 for a session lasting 60 minutes.). In this control experiment, half of the trials contained upright faces and the other half contained inverted faces. We expected to replicate our results in Experiment 3 with upright faces and demonstrate a null result with inverted faces where lower-level image features were kept while the high-level integration of face features was disrupted. Also we have added in 20 catch trials, including 10 blank trials and 10 visible trials in which faces were superimposed on the colorful Mondrians and presented at full contrast to ensure visibility of the faces. These catch trials allowed us the gauge the ratio of false alarms and misses.

The results show a successful replication of the original effect in Experiment 3 (attractive vs. unattractive: 76.61% vs. 81.38 %, $t_{\text{paired}}(9) = -2.28$, $p = .04$, Figure S2, left) in the upright face condition. As expected, the effect disappeared when faces were inverted (attractive vs. unattractive: 78.74% vs. 76.56 %, $t_{\text{paired}}(9) = 0.76$, $p = .47$, Figure S2, right). Also, participants' performances on the catch trials (mean accuracy on visible and invisible catch trials: 98% and 93 %, respectively) suggest that they could differentiate between visible and invisible faces, supporting the

validity of visibility reports. Finally, post-experiment surprise rating session in this control experiment confirmed significant differences between the two categories (attractive vs. unattractive faces: 5.8 (±0.8) vs. 2.9 (±1.0), $t_{paired}(9) = 8.58$, $p < .0001$; Rating correlation across faces between Experiments 3 and this experiment: $r = 0.85$, $p < .00001$).

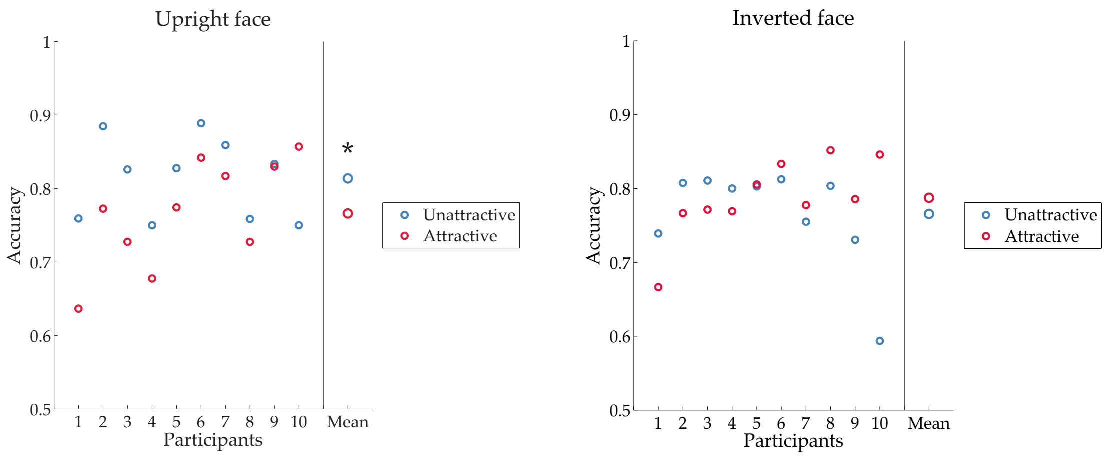

Figure S3. Accuracies on the subsequent orientation discrimination task in the control experiment of Experiment 3. For each figure, the left panel shows the accuracies subsequent to an attractive (red) or an unattractive (blue) face of individual participants, ranked by strength of the effect. The right panel shows the group mean. The results show a successful replication of Experiment 3 only when faces were upright (left) but not inverted (right).
